# Integrating Health and Care for Older People in China: What Has Been Accomplished? What is Next?

**DOI:** 10.5334/ijic.7598

**Published:** 2023-03-23

**Authors:** Linlin Hu, Ye-Fan Wang Glavin

**Affiliations:** 1 School of Health Policy and Management, Peking Union Medical College, Beijing 100730, China; 2 School of Medicine, Case Western Reserve University, Cleveland, Ohio 44106, USA; 3 IJIC Editorial Board, US

**Keywords:** integration, health, care, older people, China

In China, in addition to its rapid ageing process and large older population, the incidence of chronic disease and disability in the elderly is astonishingly high. Among the 267 million people aged 60 and over in 2021, there were about 190 million people with at least one chronic disease and 40 million living with disability [1]. This has created a huge challenge for the world’s most populous country which is also a middle-income economy in caring for its senior citizens. In response to the complex needs of the elderly and drawing from international experience, China has adopted an active ageing and healthy ageing strategy, and integrating health and care has increasingly been a policy priority for coping with population ageing in the past decade. Progress has been made while challenges remain. The experiences and lessons of China might be referential for the other middle income countries in integrating health and care for their older adults.

## Policy evolution since 2015

Integrating health and care became central government policy in 2015. Nine departments, including the current National Health and Family Planning Commission (NHFPC) and the Ministry of Civil Affairs (MCA), jointly issued the “Guiding Opinions on Promoting the Integration of Health and Elderly Care Services”. This policy document outlined the framework and principles for an integrated system of health and care. It proposed five main forms of integration: collaboration between health institutions and elderly care institutions; provision of health care services in elderly care institutions; establishment of an elderly care department in medical institutions; setting up of new institutions to provide integrated care; and provision of health care services in community elderly care settings and homes [2]. Immediately after this policy was issued, the central government launched a national pilot project for health and care integration, selecting 90 pilot cities in two batches to explore specific models, protocols and methods for the integration [3].

In the following years, supporting policies and initiatives were carried out to further promote the integration. Measures were taken to simplify the administrative procedures and create a “barrier-free”environment for the registration of new institutions providing integrated care [4,5]. In 2016, China started the piloting of long-term care insurance (LTCI) as a funding mechanism for long-term integrated care, and 15 cities across the country were selected as the first batch of national demonstration [6]. Aligning with the ongoing health care reform to strengthen primary care, especially the promotion of “contracted services of family physicians” [7] and home-based medical care [8], health and care integration at community level was vigorously promoted in policy. In 2022, the National Reform and Development Commission (NDRC), in coordination with other departments, brought out an initiative to improve the capacity of delivering health and care integrated services at the community level, with central government budget allocations to fund chained elderly care institutions in communities, basic public health services for the elderly and the training of community health and care workers [9]. In terms of quality standards, the central government published service guidelines and management guidelines for institutions providing integrated health and care services in 2019 [10] and 2020 [11]. Summarizing the experiences in pilot programs for health and care integration, a major comprehensive policy was passed in July 2022 to further push forward health and care integration [12].

After nearly a decade of efforts, providing health and care integrated services has become a dominant approach in elderly care, as well as one of the major goals for elderly care system development in China. The focus of policies has evolved from a macro-level system framework to micro-level guidelines, and from service network development to the establishment of formal institutional arrangements. A multi-level service network has gradually been formed with more and more priorities given to community and home-based service delivery. Various models of integration were piloted and implemented at different levels and in different settings [13]. The preliminary standards for service delivery and management in integrated care organizations were set up as guidelines for quality assurance. Multi-channel financing sources were established to fund the integrated services and the system has seen a notable increase in financial input both from the government and the society. By the end of 2021, there were a total of 6,492 institutions qualified to provide both health and care services, and over 90 percent of the elderly care facilities could provide medical services in different forms. In the meantime, the LTCI piloting program has expanded to 49 cities and covered about 145 million population [14].

## Unsolved issues and challenges

Despite its ambitious vision for health and care integration, enthusiasm in policy promulgation and progress in practical development, there are still some unsolved and deep-rooted problems and challenges that China has to address in the next step.

The first is fragmentation of governance. It is well acknowledged that in health and care integration, coordinated governance in the process of administration, financing and regulation of the integrated services plays a fundamental role in many countries [15,16]. However, in China, the governance structure is still fragmented. The administration of health and care falls on the MCA and the National Health Commission (NHC, renamed in 2018 from NHFPC) respectively, and the financing of the facilities and services was managed by NDRC, National Health Security Administration (NHSA) in addition to MCA and NHC. This makes it difficult to establish an aligned governance structure for integrated policy formulation, financial investment and quality regulation.

The second is inadequate and uncoordinated financing and payment. China has determined to build the LTCI as the main financing scheme for long-term elderly care [17]. However, the responsibilities of LTCI and medical insurance for financing health and care integrated services are not well defined and differentiated. In reality, medical services provided at home or in long-term care institutions are usually not fully reimbursed [18]. Given the fact that LTCI is still in its pilot stage and was remarkably limited in coverage and benefit level, the integrated services of health and care remain unaffordable for the majority of older people.

The third is lack of evidence-based protocols and standards for care integration. As China’s integrated care delivery system is in its early stage, the protocols and guidelines for care integration are extremely sparce, especially the guidelines for proactive interventions towards healthy and mildly disabled elderly [19]. Although there are international guidelines and protocols such as WHO’s ICOPE approach, they are not fully validated and implemented in China [20]. This leads to the lack of evidence-based scientific research and evaluation of current practices and models. Consequently there are unspecified quality standards and poor monitoring and evaluation of quality in practice.

The fourth is a shortage of professionals and caregivers. Although many policies seek to expand the elderly care professionals and workforce, China is still facing a huge gap in adequate supply of human resources. Health professionals, including general practitioners and nurses at community level with geriatric training are lacking [21]. Multi-disciplinary staff for integrated care, such as care managers, therapists, nutritionists, counselors, social workers, nurse assistants are very scarce [22]. It is estimated that at least 14 million nurse assistants are currently needed in China, while there are less than 0.4 million officially registered, most of whom are without formal training [23]. To improve capacity, the government has developed some educational programs to train professional care workers. However, the long training cycle, high work pressure, and low wages with few benefits have led to low enrollment in these programs [24].

## The way forward

Looking forward, China is determined to provide comprehensive and continuous health services for all the older people and promote healthy ageing. This made the integration of health and care a key component in its national strategy of healthy ageing. A policy framework has been formed and a system sketch has been established. However, barriers and weakness still existed both at the system and technical levels. We hereby recommend both policy advancement and capacity building towards the future.

With regard to policy advancement, policies and systems of financing, regulation and piloting of health and care integration should be further refined. As a potential main funding resource of integrated health and care services, the LTCI should be gradually expanded to the whole country as an independent social insurance scheme, and the benefits and coverage of LTCI should be clearly defined and differentiated from the medical insurance to have the cost of the integrated services reasonably reimbursed [25]. The regulatory system should be reformed to enable joint quality evaluation and monitoring of integrated service delivery, and unified core quality standards should be established to measure and guide quality improvement. In terms of piloting initiatives, there should be more clearly defined principles and purposes, and the effectiveness of different models and practices should be evaluated based on scientific evidence to facilitate future scale-up implementations.

With regard to capacity building, much work needs to be done in workforce training, care pathway development, and technology adoption. For senior and middle-level professionals, education programs with geriatric training should be required in medical colleges or vocational schools. For long-term care workers, more training programs should be developed with government subsidies to attract more qualified participants. In terms of care pathway, ICOPE is an effective approach to assess the functional abilities of the elderly and provide early and integrated interventions to promote autonomy and healthy ageing, which can be validated, adapted and utilized in China. Health and care integration also benefits from new technologies, such as tele-medicine, intelligent devices and big data. Setting up safe and favorable environment with the application of new technologies will greatly improve the nation’s ability to provide integrated person-centered care for the world’s largest aged population [26].

## Conclusion

In response to the pressing challenge brought by an aging population, China has chosen to provide integrated health and care services for its older people. The integration is a top-down approach, with the central government setting up goals and principles, and designating pilots and demonstrations in local areas. Along with the development in practice, supporting policies have been gradually established and refined. Despite remarkable progress in the past eight years, there are still some underlying issues and problems which need to be addressed. The financing and payment of the integrated services should be addressed by coordination of the medical insurance and the emerging LCTI with focus on total care and total cost. The effectiveness of the current models of integration should be evaluated on a scientific basis, and evidence-based care pathways or protocols should be established. In this process, integrated care should be increasingly directed to community level with a more proactive and healthy aging approach.
